# Experiences of childhood emotional maltreatment and emotional intelligence in young men

**DOI:** 10.3389/fpsyt.2026.1755465

**Published:** 2026-03-05

**Authors:** Thomas Suslow, Michael Rufer, Anette Kersting, Dennis Hoepfel

**Affiliations:** 1 Department of Psychosomatic Medicine and Psychotherapy, University of Leipzig Medical Center, Leipzig, Germany; 2 Department of Psychiatry, Psychotherapy and Psychosomatics, Psychiatric University Hospital Zurich, Zurich, Switzerland; 3 Center for Psychiatry and Psychotherapy, Hospital Zugersee Triaplus, Oberwil-Zug, Switzerland

**Keywords:** childhood trauma, emotion management, emotion perception, emotion understanding, emotion use, emotional abuse, emotional neglect, trait emotional intelligence

## Abstract

**Background:**

Long-term cognitive-affective impairments could be a significant outcome of childhood maltreatment. According to trait models of emotional intelligence, experiential abilities (emotion perception and thought facilitation through emotion) are distinguished from strategic abilities (understanding and managing emotion). In a previous study including only women with adverse childhood experiences, childhood emotional neglect was found to be linked to a diminished capacity to understand own feelings in adulthood. In the current study, relationships between childhood maltreatment experiences and trait emotional intelligence in adulthood were investigated in a sample of men.

**Materials and methods:**

The sample comprised 97 young men maltreated during childhood. The Childhood Trauma Questionnaire (CTQ) was administered to identify a history of childhood trauma. The Self-Rated Emotional Intelligence Scale (SREIS) was used to assess trait emotional intelligence. Tests measuring verbal intelligence, cognitive flexibility, trait anxiety, and depressive symptoms were also administered.

**Results:**

Emotional neglect showed a negative correlation with the SREIS subscale Managing emotion (self). No other CTQ scale was correlated with the SREIS. Depressive symptoms predicted poor emotion management. Regression analysis revealed that emotional neglect was a predictor of managing one’s emotions independent of verbal intelligence, cognitive flexibility, trait anxiety, and depressive symptoms.

**Discussion:**

In men, experiences of emotional neglect during childhood but not of other maltreatment types seem to be associated with a diminished ability to manage one’s emotions in adulthood. The present findings support the idea of a strategic emotional intelligence vulnerability following emotional neglect.

## Introduction

Child maltreatment, a form of harm by a parent or caregiver, can include physical, emotional, and sexual abuse (acts of commission), and physical and emotional neglect (acts of omission), which negatively affects a child’s development and health (1). While physical neglect during childhood refers to material and safety-related deficiencies (e.g., failures by caregivers to provide adequate food, clothing, shelter, or supervision), emotional neglect concerns the lack of affection and psychological support (e.g., denial or non-fulfillment of the child’s emotional needs for love, validation, encouragement, and sense of belonging). Childhood trauma is related to an increased likelihood of being diagnosed with a multiplicity of somatic and mental illnesses in adulthood, for example cardiovascular (2) and respiratory diseases (3), mood and anxiety disorders (4), and dissociative pathology (5). Experiences of maltreatment during childhood are associated with lower social functioning and poorer interpersonal relations in adulthood (6). Child maltreatment is a common problem worldwide (7). A recent national survey showed that in Germany 18% of young adults were victims of childhood maltreatment (8). In the latter study, respondents most frequently reported having suffered from emotional neglect, followed by physical neglect, emotional abuse, physical abuse, and sexual abuse. Child maltreatment can lead to lasting alterations in how individuals perceive, process, and regulate emotions, impacting socio-emotional development (9–12).

Children start to develop emotional competencies, i.e., knowledge of one’s own and other’s emotions and the regulation of emotional expressiveness and experience, to a large extent within close attachment relationships – with parents or other caregivers (13–16). They learn these competencies through a combination of direct teaching and everyday experiences, by observing and imitating adults, and engaging in activities like social interactions, play, and storytelling. Experiences of neglect and abuse can disturb the development of emotional competencies, resulting in alexithymic personality characteristics, i.e., difficulties in identifying and expressing emotions (17, 18). Recent meta-analyses demonstrate that experiencing emotional abuse, emotional neglect, and physical neglect during childhood is associated with impairments in identifying and describing emotions in adulthood (19, 20). Child maltreatment has also been found to be related to poor emotion regulation capacities, increased emotional suppression and lower capacity to implement cognitive reappraisal strategies in affected adults (21–23). Emotionally maltreated children lack positive role models for developing effective emotion regulation strategies (24). Emotional abuse and neglect in childhood may have more damaging consequences for a child’s socio-emotional development than physical or sexual abuse (25–27). Why emotional abuse and neglect could be particularly harmful may be due to their more direct impact on the emotion processing system. In contrast to previous research, which emphasized alexithymia and emotional dysregulation as cognitive-affective outcomes of childhood maltreatment, the impact on emotional intelligence remains less explored.

The concept of *emotional intelligence* refers to a number of capacities in dealing with emotions (28): recognizing and labeling feelings, understanding that emotions drive behavior, and using emotional information to make effective decisions and guide thinking. Emotional intelligence models are typically categorized into three groups, i.e., ability, trait, and mixed models, depending on whether they assess emotional intelligence as a cognitive skill through performance tests or as a collection of self-reported traits (29). Ability-based measures of emotional intelligence are tests of maximal performance (30). According to trait models, emotional intelligence refers to typical self-perceptions concerning the ability to recognize, use and manage emotions and typical emotion-related behavior tendencies, which can be measured by self-report questionnaires (31, 32). The emotional intelligence construct of mixed models is broader than that of trait models and can include, for example, personality factors (such as optimism and assertiveness), self-awareness, and empathic skills (33). Mayer and Salovey (34) proposed an influential distinction between *experiential* abilities of emotional intelligence (i.e., emotion perception and thought facilitation through emotion) and *strategic* abilities (i.e., emotion understanding and emotion management in oneself and others (35)). These two groups of abilities represent different levels of processing emotion, with experiential relating to lower-order (or rapid perceptual) abilities and strategic relating to higher-order (or conscious) processes of emotional reasoning and control (36, 37). Emotional intelligence is associated with the use of more efficient coping mechanisms for stress and negative affect (38, 39) and a reduced vulnerability to mental health issues like depression and anxiety (40, 41). Recognition and verbalization of one’s own emotions can attenuate negative emotional experiences (42). Identification and sharing of other people’s emotions can strengthen social bonds (43, 44).

The emotional intelligence facet of emotion management is closely related to the concept of emotion regulation but they can be at least partially differentiated from each other at a theoretical level. In emotion research, emotion regulation often refers to cognitive and behavioral processes that serve to control, modify, and influence one’s own emotional states (45, 46). From this perspective, emotion regulation constitutes the specific, often short-term, strategies and processes used to alter emotional trajectories, e.g., emotion suppression, distraction or cognitive reappraisal (47). In contrast, emotion management within the concept of emotional intelligence refers to a broader, higher-order competence to manage emotions in oneself and others to achieve goals or desired outcomes and facilitate flexible and effective regulation of emotional responses and decision-making (34, 48). Individuals with emotion management competence show high ability to choose appropriate or helpful strategies for handling distressing or otherwise emotionally impactful situations (30). The emotional intelligence facet of emotion management focuses on individual differences in the capacity to manage emotions, whereas emotion regulation is often based on process models focusing on how to regulate.

Previous studies on childhood maltreatment and emotional intelligence have focused on how emotional intelligence acts as a mediator between childhood maltreatment and psychological outcomes like depression, anxiety, and life satisfaction (49–51). In these studies, overall childhood maltreatment severity showed negative correlations of small to medium size with trait emotional intelligence in samples of university students (49–51). In analyses that considered the specific form of child maltreatment, negative correlations of small to medium size were found between emotional abuse and emotional neglect and total trait emotional intelligence (52). To our knowledge, only one study has investigated the question of which types of childhood experiences are related to which facets of emotional intelligence (53). In a sample of women with various maltreatment experiences during childhood, emotional neglect was negatively correlated with the emotional intelligence facet of understanding emotion. It was concluded that experiences of emotional neglect during childhood but not of other maltreatment types could be linked with a decreased ability to understand emotional states in adulthood. These findings indicate that early emotional neglect might have an impact on strategic (but not on experiential) emotional abilities and that emotional neglect could have a greater impact on the development and expression of emotional intelligence than emotional abuse. Because the study exclusively examined women, the generalizability of its results is limited. In fact, there is evidence that experiences of childhood maltreatment can have different adverse effects on emotional functioning in men and women. Women exposed to childhood trauma are more likely to develop depression or anxiety disorders (54, 55), whereas men with childhood trauma exposure are more likely to engage in risk-taking, violent or aggressive behaviors (56).

In the current study, we investigated the relationship of childhood maltreatment with trait emotional intelligence in a sample of men maltreated in childhood. Based on our previous results observed in a sample of maltreated women (53), we hypothesized that emotional neglect experiences are negatively correlated with the emotional intelligence facet understanding emotion. The main focus of our study was on the relations between the emotional forms of childhood maltreatment and the specific facets of emotional intelligence. We controlled participants’ verbal intelligence, cognitive flexibility, depressive symptoms, and trait anxiety, which represent relevant variables in the research context. Childhood maltreatment can be associated with impairments in verbal intelligence and executive functions in adulthood (57, 58) and with heightened anxiety and depressive symptoms (55, 59). The procedures and statistical analysis methods used in the present study were identical to those administered in our previous investigation (53), allowing a direct comparison between them.

## Materials and methods

### Participants

Men were recruited as study participants via online advertisements and public notices posted in student halls of residence, canteens, libraries, and public buildings of the University of Leipzig. In our advertisements, we mentioned the main forms of possible abuse (emotional, physical, and sexual) and neglect (emotional and physical). To be included, participants must have self-reported at least one of these forms of maltreatment in their childhood. The self-reported information was confirmed by the CTQ data: according to the severity criteria of Bernstein and Fink (60) all of our study participants had at least one type of maltreatment experience at level 2 (“low to moderate”) during childhood. The final sample included 97 young men with a mean age of 25.24 years (SD = 4.26; range: 18–35). The majority of participants were university students (n = 57). The other participants were working (n = 22), unemployed (n = 13), or in vocational training (n = 4) (one participant did not provide information). Individuals interested in our study were interviewed via telephone to check inclusion/exclusion criteria. Male biological sex, native German language, and age between 18 and 35 years were inclusion criteria. Exclusion criteria were presence of a diagnosed mental or neurological disorder or use of psychotropic medication. Men with psychiatric, neurological, or psychotherapeutic treatments were excluded. The ethics committee of the University of Leipzig (Medical School) approved the study procedure. From all participants written informed consent was obtained. Participants were financially reimbursed for their time.

### Questionnaires and tests

Maltreatment experiences during childhood were assessed using the Childhood Trauma Questionnaire (CTQ (60), German version (61)). The CTQ is a 25-item retrospective self-report measure with five subscales: physical abuse, emotional abuse, sexual abuse, physical neglect, and emotional neglect. Each subscale of the CTQ consists of 5 items. Four categories of severity for each maltreatment type and the total trauma experience have been proposed (60): None (or minimal); Low (to moderate); Moderate (to severe); and Severe (to extreme). Internal consistencies (Cronbach’s alpha) were satisfactory for the CTQ scales with the exception of the physical neglect scale: 0.84 for the total CTQ, 0.78 for physical abuse, 0.74 for emotional abuse, 0.90 for sexual abuse, 0.73 for emotional neglect, and 0.47 for physical neglect. Klinitzke et al. (62) also found low internal consistency for physical neglect and cautioned against the use of the scale.

The Self-Rated Emotional Intelligence Scale (SREIS (63), German version (64)) was applied to measure emotional intelligence. The SREIS consists of five subscales assessing four facets of emotional intelligence: perception, use, understanding, and managing of (one’s own and other people’s) emotion. The items are assessed on a scale from 1 (inaccurate) to 5 (accurate). The scale Perceiving emotion comprises four items related to the perception and identification of emotions expressed by others (e.g., “I am aware of the nonverbal messages other people send”). The scale Use of emotion consists of three items related to the ability to harness emotions, which can assist problem-solving processes and reasoning (e.g., “When making decisions, I listen to my feelings to see if the decision feels right”). The scale Understanding emotion has four items, which relate to the ability to verbalize and understand emotions (e.g., “I have a rich vocabulary to describe my emotions”). The scale Managing emotion comprises four items relating to the ability to control and change one’s emotional responses (e.g., “I know how to keep calm in difficult or stressful situations”). The scale Social management has four items referring to the ability to modify and enhance emotional states in other people (e.g., “I know the strategies to make or improve other people’s moods”). By summing the subscale scores a total emotional intelligence score can be calculated. In our study, Cronbach’s alpha was 0.54 for Perceiving emotion, 0.86 for Use of emotion, 0.87 for Understanding emotion, 0.74 for Managing emotion, 0.81 for Social management, and 0.81 for the total score.

The State-Trait Anxiety Inventory (STAI (65); German version (66)) can assess both state anxiety and trait anxiety (a person’s general tendency to be anxious and evaluate situations as threatening). In our study, only the trait version was applied. The STAI comprises 20 items that are assessed on a 4-point scale, with total scores ranging from 20 to 80. Cronbach’s alpha for the STAI trait was 0.90 in our sample.

The Beck Depression Inventory is a 21-item, self-report questionnaire used to measure the presence and severity of depressive symptoms, assessing various physical, emotional, and cognitive manifestations of depression (BDI-II (67); German version (68)). The items have a set of four possible answers, ranging in intensity. The total scores can range from a minimum of 0 to a maximum of 63. In the present sample, Cronbach’s alpha for the BDI-II was 0.89.

To assess participants’ cognitive flexibility Part B of the Trail Making Test (TMT-B (69)) was administered. In this paper-and-pencil test, participants are asked to connect numbers and letters in ascending order. The total time required to solve the task serves as an indicator of cognitive flexibility.

The Multiple-choice vocabulary intelligence test (Mehrfachwahl-Wortschatz-Intelligenztest, MWT-B (70)) is a measure of verbal intelligence. The MWT-B comprises 37 items and has no time restrictions. Each item consists of a single real German word and four pronounceable pseudo-words. Participants have to identify the real word. Each correctly recognized word is awarded one point. The total score is used to calculate an intelligence quotient.

### General procedure

Study participants were individually tested in a quiet room at the Department of Psychosomatic Medicine and Psychotherapy at the University of Leipzig. After filling in our sociodemographic questionnaire, a series of questionnaires and tests were administered in a fixed order: CTQ, BDI-II, STAI, MWT-B, SREIS, and TMT-B. Completing the test instruments took about 45 minutes.

### Statistical analysis

Normality of variable distribution was analyzed with the Shapiro-Wilk test. As violations of the assumption of normal distribution were observed for the majority of the variables (*p*s < 0.05; except for the CTQ scale emotional neglect, the SREIS total scale, the SREIS subscales understanding emotion and social management, and the STAI) we administered primarily nonparametric statistical tests. To analyze differences in severity of childhood maltreatment types reported in our sample we used the Friedman test. Wilcoxon tests were administered *post-hoc*. The relationships between childhood maltreatment types, emotional intelligence facets, verbal intelligence, cognitive flexibility, depression, and trait anxiety were examined using Spearman rank correlation. In our first step, we tested whether emotional abuse and emotional neglect are related to (total) emotional intelligence. In our second step, we investigated whether emotional abuse and emotional neglect are related to specific facets of emotional intelligence. This is the main research question of the present study. To correct for multiple testing, an adjusted p-level of 0.005 (two-tailed) was used in the correlation analyses between CTQ and SREIS, i.e., we divided the standard significance level of *p* = 0.05 by ten (2 emotional forms of childhood maltreatment x 5 facets of emotional intelligence). This p-level corresponds exactly to the one used in our previous study (53). Most correlation analyses were carried out to control confounders and to identify factors associated with facets of emotional intelligence or childhood maltreatment. Hierarchical regression analysis was performed for those facets of emotional intelligence that showed correlations with childhood maltreatment, to test whether these relationships remain significant after adjusting the effects of other relevant variables, i.e., depression, trait anxiety, verbal intelligence, and cognitive flexibility. Regression analysis is relatively robust against non-normality but regression residuals should follow a normal distribution. Multicollinearity of variables was assessed using tolerance and variance inflation factor (71). To test for serial autocorrelation the Durbin-Watson test was administered. Durbin-Watson values around 2 indicate that there is no serial autocorrelation. Statistical analyses were calculated using SPSS software version 29.0 (IBM Corp., Armonk, NY, USA).

## Results

### Severity of childhood maltreatment

Significant differences in severity between childhood maltreatment types were observed in our sample, χ^2^ (4) = 246.69, *p* < 0.001 (descriptive statistics for the CTQ are presented in **Table 1**). According to results of Wilcoxon tests, severity of all childhood maltreatment types differed significantly from each other with one exception (emotional neglect > emotional abuse > physical neglect = physical abuse > sexual abuse, *p*s <.001). This means that the severity of physical neglect did not differ from the severity of physical abuse in our sample. Moreover, we classified study participants into the four categories of severity for each trauma type and the total trauma experience as suggested by Bernstein and Fink (60) (see **Supplementary Table 1** for details). All participants were characterized by at least one form of childhood maltreatment experience - at least of low severity. Almost half of the participants reported to have experienced severe emotional neglect (44%). Approximately one quarter reported severe emotional (24%) or physical abuse (26%). However, more than half of the participants reported no experience of physical abuse (59%) and the majority reported no experience of sexual abuse (80%).

**Table 1:** Descriptive statistics of and Spearman rank correlations between CTQ scales and SREIS scales (N = 97).

Variable	CTQ total	CTQ EA	CTQ PA	CTQ SA	CTQ EN	CTQ PN	Mean	*SD*
SREIS total	-.08	.04	.00	.05	-.27	-.13	63.40	9.66
PE	.03	.08	.04	-.03	-.06	-.01	15.21	2.48
UsE	-.03	.02	-.04	.03	-.07	-.03	9.54	3.06
UnE	-.11	.00	-.07	.00	-.16	-.14	12.90	3.73
ME	-.23	-.18	-.12	-.05	-.29**	-.17	12.11	3.40
SM	.09	.12	.16	.09	-.14	.02	13.65	3.34
*Mean*	54.21	12.66	8.72	6.35	16.92	9.56		
*SD*	12.88	4.72	4.58	3.50	3.66	3.01		

**p <.005 (two-tailed).

CTQ, Childhood Trauma Questionnaire; CTQ-EA, scale emotional abuse; CTQ-PA, scale physical abuse; CTQ-SA, scale sexual abuse; CTQ-EN, scale emotional neglect; CTQ-PN, scale physical neglect; SREIS, Self-Rated Emotional Intelligence Scale; PE, perceiving emotion scale; UsE, use of emotion scale; UnE, understanding emotion scale; ME, managing emotion (self) scale; SM, social management scale.

### Relationships between CTQ, SREIS, MWT-B, TMT-B, BDI-II, and STAI

**Figure 1:**
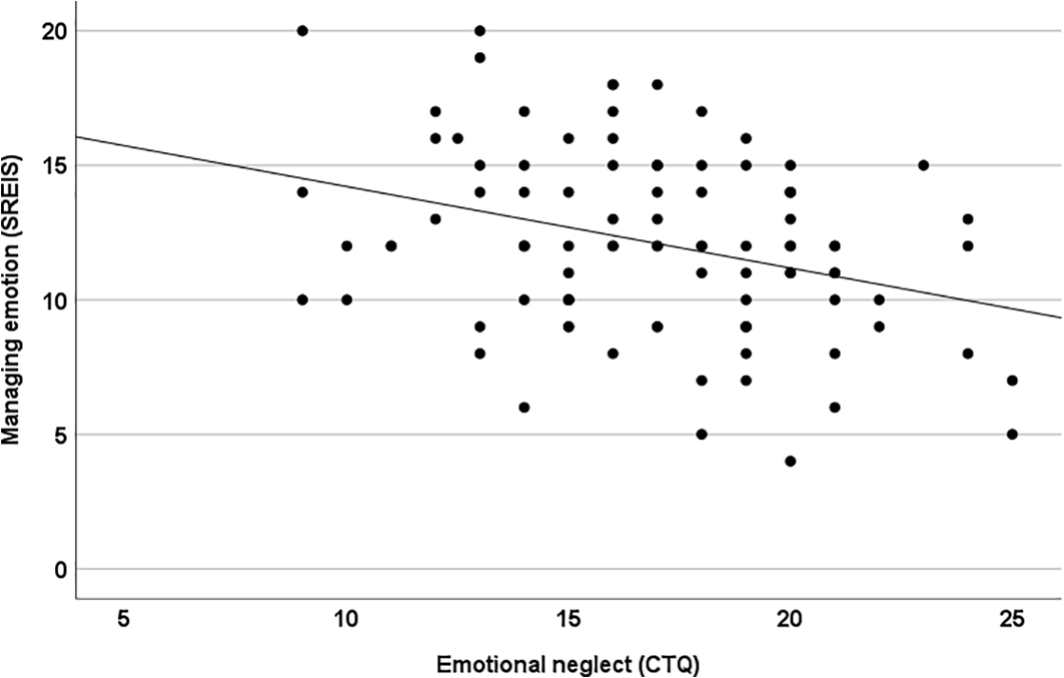
The scatterplot shows the correlation between emotional neglect (CTQ) and managing emotion (self) (SREIS) (r_s_=-29, p < .005).

We present the means with standard deviations for the SREIS in **Table 1**. Emotional neglect (CTQ) was negatively correlated with the SREIS subscale Managing emotion (self) (see **Table 1**; **Figure 1**). This correlation was of medium size. There were no other significant correlations between CTQ and SREIS.

**Table 2** shows the means with standard deviations for the MWT-B, TMT-B, BDI-II, and STAI. The total score of the CTQ and the subscales Emotional abuse, Emotional neglect, and Physical neglect were positively correlated with the BDI-II. No other significant correlations of the CTQ were found with the MWT-B, TMT-B, BDI-II, and STAI.

**Table 2:** Descriptive statistics of and Spearman rank correlations between CTQ scales and other self-report scales (BDI-II and STAI) and tests (MWT-B and TMT-B) (N = 97).

Variable	CTQ total	CTQ EA	CTQ PA	CTQ SA	CTQ EN	CTQ PN	Mean	*SD*
MWT-B IQ	.02	.01	.00	-.05	.02	.12	106.67	10.07
TMT-B	.00	-.11	.06	.18	-.07	-.06	67.42	22.85
BDI-II	.26*	.23*	.15	.08	.28**	.24*	14.93	9.51
STAI	.04	.16	-.01	.07	-.04	-.08	46.93	5.25

* *p* <.05 (two-tailed), ** *p* <.01 (two-tailed).

CTQ: Childhood Trauma Questionnaire; CTQ-EA: scale emotional abuse; CTQ-PA: scale physical abuse; CTQ-SA: scale sexual abuse; CTQ-EN: scale emotional neglect; CTQ-PN: scale physical neglect; MWT-B IQ: Multiple-choice vocabulary test version B, intelligence quotient; TMT-B: Trail-Making-Test version B; BDI-II: Beck Depression Inventory; STAI: State-Trait Anxiety Inventory, trait version. .

The total score of the SREIS showed a negative correlation with the BDI-II (see **Supplementary Table 2**). The SREIS subscales Understanding emotion, Managing emotion, and Social management were also negatively correlated with the BDI-II. There were no other significant correlations of the SREIS scales with the MWT-B, TMT-B, BDI-II, and STAI.

A regression model for managing emotion was calculated to test whether emotional neglect is a predictor independent from verbal intelligence (MWT-B), cognitive flexibility (TMT-B), depressive symptoms (BDI-II), and trait anxiety (STAI). In the first step of the regression analysis, variance in managing emotion was significantly explained by BDI-II scores, with individuals reporting more depressive symptoms having lower Managing emotion scores (see **Table 3**). Entering emotional neglect in step two significantly increased the predictive value of the model. Emotional neglect was a significant negative predictor of managing emotion. The model’s regression residuals were normally distributed (Shapiro-Wilk *W* = 0.98, df = 97, *p* = 0.36). The Durbin-Watson test yielded *d* = 1.99. Tolerance and VIF values indicate no multicollinearity in the model (see **Table 3**).

**Table 3:** Hierarchical regression predicting managing emotion self (SREIS) in two steps by verbal intelligence (MWT-B IQ), cognitive flexibility (TMT-B), depressive symptoms (BDI-II), trait anxiety (STAI), and emotional neglect (CTQ) (N = 97). .

Step	Predictor	Coefficients				Multicollinearity		Model	
		β [with 95% confidence interval]	Beta	*T*	Sig. (*p*)	Tol.	VIF	R^2^	∆R^2^
Step1	MWT-B IQ	-.051 [-0.114,0.011]	-.152	-1.63	.105	.99	1.01	.216	–
	TMT-B	-.015 [-0.042,0.013]	-.100	-1.08	.284	.99	1.01		
	BDI-II	-.149 [-0.220,-0.079]	-.418	-4.19	<.001*	.86	1.17		
	STAI	-.026 [-0.155,0.102]	-.041	-0.41	.684	.85	1.17		
Step2	MWT-B IQ	-.043 [-0.104,0.018]	-.126	-1.39	.168	.97	1.02	.270	.053*
	TMT-B	-.018 [-0.044,0.009]	-.119	-1.32	.191	.99	1.01		
	BDI-II	-.121 [-0.193,-0.049]	-.338	-3.33	.001*	.78	1.28		
	STAI	-.054 [-0.180,0.073]	-.083	-0.84	.401	.83	1.20		
	CTQ-EN	-.227 [-0.402,-0.052]	-.244	-2.58	.012*	.89	1.12		

β = unstandardized regression coefficient, Tol. = Tolerance, VIF = Variance Inflation Factor.

* *p* ≤.05 (two-tailed).

## Discussion

In this study, we examined the relationships of childhood maltreatment types with facets of trait emotional intelligence in a sample of young men with various adverse childhood experiences. Our results indicate that, in men, emotional neglect is negatively linked to the ability to manage one’s own emotions. Considering the measurement construct of the SREIS subscale, it can be formulated more precisely that emotional neglect was found to be associated with lower competence to downregulate or block negative emotions in stressful situations. This relation was independent of trait anxiety, depressive symptoms, verbal intelligence, and cognitive flexibility. Depressive symptoms were found to be associated with poor emotion management. The latter result is consistent with previous research showing that depressed patients use more frequently inefficient or maladaptive strategies when regulating emotions and have difficulties effectively implementing adaptive strategies (72, 73). We found no support for the hypothesis that emotional neglect experiences are related to the emotional intelligence facet understanding emotion. In a previous investigation with women, who had been maltreated during childhood, emotional neglect was correlated with a decreased capacity to understand and verbalize emotional states (53). This pattern of results across studies suggests, on the one hand, that it is primarily emotional neglect that could be related to emotional intelligence in adulthood. On the other hand, it indicates that the relationships between emotional child neglect and emotional intelligence may differ for the two sexes. However, it must be acknowledged that this assumption about sex differences is not based on a direct comparison (using within-sample tests), but on a comparison of results between studies. Summing up, it can be concluded that for both men and women emotional neglect was found to correlate with a strategic emotional intelligence ability, i.e., emotion management or emotion understanding.

Our data suggests that, in men, early experiences of emotional neglect, i.e., having received little emotional attention and support from caregivers might be related to a restricted ability to control and manage emotional reactions in adulthood. The present finding indicates possible relationships between emotional neglect experiences during childhood and alterations in individual differences in the regulatory handling of emotions and thereby may shed light on potential deficits in a specific strategic aspect of emotional intelligence that go beyond the existing research on emotion regulation, which is often process-oriented. As stated previously, emotion management within the concept of emotional intelligence refers to a higher-order competence to manage emotions in oneself (and others) in a flexible and context-dependent way to achieve goals or desired outcomes (34, 48).

Morris et al. (74) presented a tripartite model on the impact of the family on children’s emotion regulation. According to the authors, parents affect children’s emotion regulation ability in three ways: emotion-related parenting practices like teaching regulation strategies, observation of parents’ emotion regulation (e.g., modeling, and emotion contagion), and emotional climate of the family (e.g., parent-child attachment, and positive emotionality expressed in the family) (75). Experiences of emotional neglect in childhood may impair emotion regulation abilities via all three mechanisms described. Emotionally neglected children may lack parents as teachers, models, and positive attachment figures. Longitudinal data has shown that greater childhood neglect is associated with greater problems in emotion regulation at age 14 indicating that neglect during childhood predicts the initial levels of emotion regulation difficulties in early adolescence (76). Compared to girls, parents use more active and instrumental strategies for regulating emotions among boys (16, 77). This parenting behavior could be due to the fact that during childhood boys show more externalizing emotions like anger than girls (78). Against this background, one could speculate whether experiences of emotional neglect in boys might be more strongly related to limitations in emotion regulation skills than in girls. More externalizing children might be harmed most by absent or poor responses to their emotions on the part of parents. However, based on our cross-sectional data and in the absence of a direct interaction test (emotional neglect x sex), no sex-specific developmental claims should be made.

According to the present preliminary findings and those of Suslow et al. (53), emotional neglect could have a greater effect on the development and expression of emotional intelligence than emotional abuse. The absence of role models in the area of ​​emotion-related learning might have a more negative influence on the development of emotion knowledge and emotion management than models that maltreat their children by using emotions to scare, humiliate, manipulate or hurt them. If emotional exchange between child and caregiver still occurs (albeit very problematic) children can perceive other’s emotions and develop own reactions that can become objects of reflection and regulation. Emotional care and attention received from caregivers during childhood could have more importance for the development of higher-order than of lower-order (i.e., experiential) processes of emotional intelligence.

It can be helpful in interpretating the differential correlation findings for men and women to take a closer look at the distributions and characteristics of childhood trauma experiences and emotional intelligence in the present male sample (compared to our previous female sample). Considering the means of the CTQ scales the men of our sample were primarily characterized by experiences of emotional neglect and emotional abuse during childhood, followed by physical neglect and physical abuse, whereas few experiences of sexual abuse were reported. The present male sample had significantly lower emotional abuse (Cohen’s *d* = .71) and emotional neglect scores (*d* = .43) than the previous female sample (53) (see for statistical details **Supplementary Table 3**). These results are for emotional abuse in line with expectations, as in a recent young adult sample of the German population (8) women were at a higher risk for emotional abuse than men. Men (in the present sample) showed lower emotion use (*d* = .33) and social management of emotion scores (*d* = .31) and higher scores for management of own emotions (*d* = .38) than the women in our previous sample. Not surprisingly, men reported less trait anxiety (*d* = .40) than women. No group differences were found for depressive symptoms, verbal intelligence, and cognitive flexibility. So, there are differences between the sexes in the extent of experienced emotional neglect and the ability to manage one’s emotions, but, according to the results of Levene tests, variances of the CTQ und SREIS scores were equal for men and women (with one exception: physical abuse, *p* <.05). This means that differential correlations between emotional neglect and strategic emotional intelligence facets for men and women should not be due to low variance of scores in one of the samples.

There are several limitations of the current study that must be noted. We assessed adverse childhood experiences only retrospectively by a self-report questionnaire. Even though the CTQ is a validated instrument for assessing childhood trauma, it does not include information about the time adversity occurred and it focuses on maltreatment experiences within the family. Test instruments like the Maltreatment and Abuse Chronology of Exposure scale (79) provide more detailed information on intensity, duration, frequency, and age of first exposure to childhood maltreatment. Given the poor internal consistency of the CTQ subscale physical neglect null findings involving physical neglect should not be overinterpreted. Internal consistency was also low for the SREIS scale Perceiving emotion. Against this background, the observed non-correlations of this scale with the CTQ must be interpreted with caution. Poor reliability limits the ability to detect true associations and can in part explain our null finding for the experiential emotional intelligence facet Perceiving emotion. Thus, the lack of correlations for emotion perception (and physical neglect) could be due to measurement constraints rather than true null effects. In our study, we focused on trait emotional intelligence (i.e., self-perceptions of abilities and typical behaviors). Since trait emotional intelligence is only weakly correlated with ability emotional intelligence (80), it is a major task of future research to investigate the links of childhood maltreatment with emotional intelligence as assessed by ability tests. As our study design was cross-sectional our correlational data does not allow us to infer causality. The generalizability of our findings is limited by the fact that we included young, predominantly well-educated men, who did not suffer from mental or neurological disorders and did not use psychotropic medication. Our results cannot be generalized to older people or clinical populations where deficits caused by maltreatment might be more pronounced. The restricted range of psychopathology in our study sample could have attenuated associations and might have biased our findings toward higher emotion management capacity. A further limitation of this study is that the severity of the five childhood maltreatment types examined varied significantly, with sexual abuse being rarely reported in our sample.

## Data Availability

The raw data supporting the conclusions of this article will be made available by the authors, without undue reservation.
