# Supplementary material for: Experiences of childhood emotional maltreatment and emotional intelligence in young men
Source: Front Psychiatry. 2026 Mar 5;17:1755465. doi: 10.3389/fpsyt.2026.1755465 (PMC12999840; doi:10.3389/fpsyt.2026.1755465)
Supplement: Supplementary file 1 [file Table1.docx]

Supplementary Table 1: Distribution of study participants’ CTQ subscale and total scores ​​across categories of childhood maltreatment severity (classification as proposed by Bernstein and Fink, 1998).

| CTQ | None | Low | | Moderate | Severe |
| --- | --- | --- | --- | --- | --- |
| Emotional abuse | 21 | 30 | 23 | | 23 |
| Physical abuse | 57 | 5 | 10 | | 25 |
| Sexual abuse | 78 | 5 | 7 | | 7 |
| Emotional neglect | 3 | 22 | 29 | | 43 |
| Physical neglect | 26 | 29 | 26 | | 16 |
| Total CTQ | 3 | 45 | 33 | | 16 |
|  | | | | | |
