# Supplementary material for: Experiences of childhood emotional maltreatment and emotional intelligence in young men
Source: Front Psychiatry. 2026 Mar 5;17:1755465. doi: 10.3389/fpsyt.2026.1755465 (PMC12999840; doi:10.3389/fpsyt.2026.1755465)
Supplement: Supplementary file 2 [file Table2.docx]

**Table S2.** Spearman rank correlations between SREIS and other self-report scales (BDI-II and STAI) and tests (MWT-B and TMT-B) (N = 97).

| Variable | SREIS total | SREIS PE | SREIS UsE | SREIS UnE | SREIS ME | SREIS SM |
| --- | --- | --- | --- | --- | --- | --- |
| MWT-B IQ | .02 | -.04 | .06 | .18 | -.11 | -.08 |
| TMT-B | .06 | .11 | .13 | -.04 | -.05 | .07 |
| BDI-II | -.26* | .09 | .05 | -.23* | -.41*** | -.29** |
| STAI | -.04 | -.08 | .17 | -.07 | -.17 | .00 |

* *p* < .05 (two-tailed), ** *p* < .01 (two-tailed), *** *p* < .001 (two-tailed)

SREIS: Self-Rated Emotional Intelligence Scale; PE: perceiving emotion scale; UsE: use of emotion scale; UnE: understanding emotion scale; ME: managing emotion (self) scale; SM: social management scale; MWT-B IQ: Multiple-choice vocabulary test version B, intelligence quotient; TMT-B: Trail-Making-Test version B; BDI-II: Beck Depression Inventory; STAI: State-Trait Anxiety Inventory, trait version.
