# Supplementary material for: Experiences of childhood emotional maltreatment and emotional intelligence in young men
Source: Front Psychiatry. 2026 Mar 5;17:1755465. doi: 10.3389/fpsyt.2026.1755465 (PMC12999840; doi:10.3389/fpsyt.2026.1755465)
Supplement: Supplementary file 3 [file Table3.docx]

**Table S3.** Comparison between the present male sample and the female sample of Suslow et al. (2025) concerning age, childhood maltreatment scores, emotion intelligence scores, trait anxiety, depressive symptoms, intelligence, and cognitive flexibility. Descriptive statistics (means and SDs) and results of unpaired *t*-tests.

| ***Variable*** | **Men (n = 97)**  **Mean SD** | | **Women (n = 97)**  **Mean SD** | | ***t*** | ***p*** |
| --- | --- | --- | --- | --- | --- | --- |
| Age (years) | 25.24 | 4.26 | 23.19 | 3.38 | 3.71 | <.001*** |
| CTQ total score | 54.21 | 12.88 | 58.89 | 11.10 | -2.71 | .007** |
| CTQ-PA | 8.72 | 4.58 | 7.88 | 4.00 | 1.37 | .17 |
| CTQ-EA | 12.66 | 4.72 | 15.86 | 4.25 | -4.95 | <.001*** |
| CTQ-SA | 6.35 | 3.50 | 6.71 | 3.60 | -0.71 | .48 |
| CTQ-PN | 9.56 | 3.01 | 9.97 | 2.96 | -0.96 | .34 |
| CTQ-EN | 16.92 | 3.66 | 18.47 | 3.57 | -2.99 | .003** |
| SREIS total score | 63.40 | 9.66 | 64.03 | 9.62 | -0.45 | .65 |
| SREIS-PE | 15.21 | 2.48 | 15.51 | 3.01 | -0.75 | .45 |
| SREIS-UsE | 9.54 | 3.06 | 10.58 | 3.17 | -2.33 | .021* |
| SREIS-UnE | 12.90 | 3.72 | 12.52 | 3.76 | 0.71 | .48 |
| SREIS-ME | 12.11 | 3.40 | 10.82 | 3.29 | 2.68 | .008** |
| SREIS-SM | 13.65 | 3.34 | 14.61 | 2.86 | -2.15 | .033* |
| STAI T | 46.93 | 5.25 | 50.12 | 10.01 | -2.77 | .006** |
| BDI-II | 14.93 | 9.51 | 16.42 | 9.02 | -1.12 | .26 |
| MWT-B IQ | 106.67 | 10.07 | 106.30 | 9.47 | 0.26 | .79 |
| TMT-B | 67.42 | 22.85 | 65.51 | 26.15 | 0.55 | .59 |

* *p* < .05 (two-tailed), ** *p* < .01 (two-tailed), *** *p* < .001 (two-tailed)

CTQ: Childhood Trauma Questionnaire; CTQ-PA: scale physical abuse; CTQ-EA: scale emotional abuse; CTQ-SA: scale sexual abuse; CTQ-PN: scale physical neglect; CTQ-EN: scale emotional neglect; SREIS: Self-Rated Emotional Intelligence Scale; SREIS-PE: perceiving emotion scale; SREIS-UsE: use of emotion scale; SREIS-UnE: understanding emotion scale; SREIS-ME: managing emotion (self) scale; SREIS-SM: social management scale; STAI T: State-Trait-Anxiety Inventory, trait version; BDI-II: Beck Depression Inventory; MWT-B IQ: Mehrfachwahl-Wortschatz-Intelligenztest version B, intelligence quotient; TMT-B: Trail-Making-test version B.
